# The Midazolam RAMPART Study Medical Records Project: A Unique Use of Real-World Data in a Complex Collaborative Partnership to Support a New Drug Application

**DOI:** 10.1007/s43441-022-00447-4

**Published:** 2022-08-20

**Authors:** Nancy A. Sherman, Robert Silbergleit, Erin M. Bengelink, Valerie Durkalski, Kevin D. Wolter

**Affiliations:** 1 Post Approval Clinical Development, Pfizer Inc., 235 East 42nd Street, Office 235/6/62, New York, NY 10017 USA; 2 Department of Emergency Medicine, University of Michigan, Ann Arbor, MI USA; 3 Department of Public Health Sciences, Medical University of South Carolina, Charleston, SC USA; 4 Pfizer Inc., Groton, CT USA

**Keywords:** Lorazepam, Midazolam, Real-world data, RAMPART, Regulatory, Status epilepticus

## Abstract

**Introduction:**

This project aimed to retrospectively obtain, review, and extract key safety data from medical records of participants enrolled in RAMPART, the NIH-supported Rapid Anticonvulsant Medication Prior to ARrival Trial of intramuscular midazolam versus intravenous lorazepam for pre-hospital treatment of status epilepticus, to support a US new drug application (NDA) for intramuscular midazolam.

**Methods:**

A collaborative partnership was established between the NDA sponsor, the RAMPART trial lead academic institution, US government agencies, and contract research organizations to retrieve, review, and extract relevant safety data from the medical records of RAMPART participants and summarize those data to include in an NDA submitted to the US Food and Drug Administration (FDA).

**Results:**

Key data in the medical records of 890 RAMPART trial participants (1020 enrollments, including 130 repeat enrollments) were reviewed and extracted into a project database. Safety events occurred in 771 (86.6%) participants, and included additional information not collected in the RAMPART trial. This database also enabled subgroup analyses based on medical history and prior/concurrent medications, building upon previous analyses according to age, sex, and race. No previously unrecognized safety patterns were identified, and no association was observed between efficacy and medical history or medication usage.

**Conclusions:**

The use of unstructured real-world retrospective medical record data can effectively support an NDA submission in place of conducting another interventional clinical trial. This retrospective medical records review and extraction of additional safety data contributed to the FDA approval of intramuscular midazolam for the pre-hospital treatment of status epilepticus in 2018.

**ClinicalTrials.gov:**

NCT00809146.

## Introduction

Midazolam is a short-acting hypnotic-sedative drug of the benzodiazepine class approved in the US since the 1980s for the treatment of insomnia and as a sedative-hypnotic for use in anesthesia [1, 2]. The randomized Rapid Anticonvulsant Medication Prior to ARrival Trial (RAMPART; NCT00809146) evaluated the efficacy and safety of intramuscular midazolam and intravenous lorazepam for the treatment of status epilepticus. RAMPART was a randomized, controlled, double-blind, double-dummy clinical trial conducted by the Neurological Emergencies Treatment Trials network and clinically coordinated by the University of Michigan (UM) with grant funding from the National Institute of Neurological Disorders and Stroke (NINDS), an institute within the National Institutes of Health (NIH). The primary efficacy outcome in RAMPART was the termination of seizure prior to arrival at the emergency department (ED), without the use of rescue medication, and the primary analysis demonstrated superiority of midazolam over lorazepam [3]. As midazolam was not approved for the treatment of status epilepticus by the US Food and Drug Administration (FDA), the study was conducted under an Investigational New Drug application [3].

Status epilepticus represents a medical emergency that can lead to permanent brain damage or death, and immediate treatment is critical to improve outcomes [4]. Although status epilepticus is relatively rare (estimated to account for < 1% of hospital admissions) [5], it can result from various medical conditions or exposure to chemical or biological poisons.

The new drug application (NDA) submission included key safety and efficacy data from RAMPART. RAMPART enrolled patients from 33 emergency medical services agencies associated with 79 US hospitals and demonstrated that intramuscular midazolam administered via autoinjector was more effective than the standard of care, intravenous lorazepam, for the treatment of status epilepticus in children and adults when administered by emergency medical personnel in the pre-hospital setting. RAMPART also showed comparable safety of midazolam and lorazepam, including the incidence of serious adverse events (SAEs) [3, 6]. The trial captured all AEs, both serious and non-serious, occurring within 24 h of drug administration, and all SAEs occurring between drug administration and the end of hospitalization. AEs were categorized using standard Medical Dictionary for Regulatory Activities (MedDRA) preferred terms and system organ classes.

The successful efficacy results from RAMPART were pivotal to the NDA submission; however, the FDA advised that more comprehensive safety, clinical, and other relevant data would be required to support the safety of midazolam for this new use and recommended that comprehensive safety information be extracted from the medical records of RAMPART participants and presented using MedDRA.

Real-world data (RWD) have typically been used for post-marketing surveillance but this situation has changed in recent years, with some instances of RWD providing support for successful NDAs [7, 8]. In 2018, the FDA unveiled their real-world evidence program, which includes comprehensive information regarding the use of RWD to support regulatory decisions [9]. More recently, in 2021 the FDA released further information to assist the industry in designing non-interventional studies and in the use of RWD to support new drug applications [10, 11]. In general, RWD can be used for both safety and efficacy data, but considerations should be taken before its use in a study. RWD is most applicable when there is a robust data source(s) with a diverse patient population, and good study design is possible with the data available.

This medical records project involved a public–private partnership that developed a detailed project plan to collect unstructured, retrospective, RWD from the medical records of RAMPART participants. The intent was to comprehensively identify and extract all safety, clinical, and other relevant data requested by the FDA and create an analyzable database to support NDA review. The medical records data were intended to complement and supplement the information from the prospective, interventional RAMPART clinical trial. However, comprehensive collection of all safety information from a patient’s medical record, rather than the focused collection of more proximate AEs and pre-specified safety outcomes, was unprecedented and required the development of a suitable methodology (henceforth called the ‘program plan’) that included the detailed processes and procedures that would be needed. Furthermore, as the FDA anticipated that further questions could arise during NDA review, they recommended gathering other potentially useful clinical data, beyond safety events; for example, vital signs, laboratory tests, imaging results, bioelectrical signals, blood gases, and blood cultures. This early communication with the FDA regarding study design and data collection is in line with their recent Guidance for Industry documents [10, 11].

Therefore, the objective of this midazolam RAMPART medical records project (MRP) was to retrospectively obtain and review the medical records of all RAMPART participants to comprehensively identify and extract safety and other clinical data, including renal and hepatic events, into an analyzable database to support NDA review of a new indication for midazolam.

## Methods

**Fig. 1:**
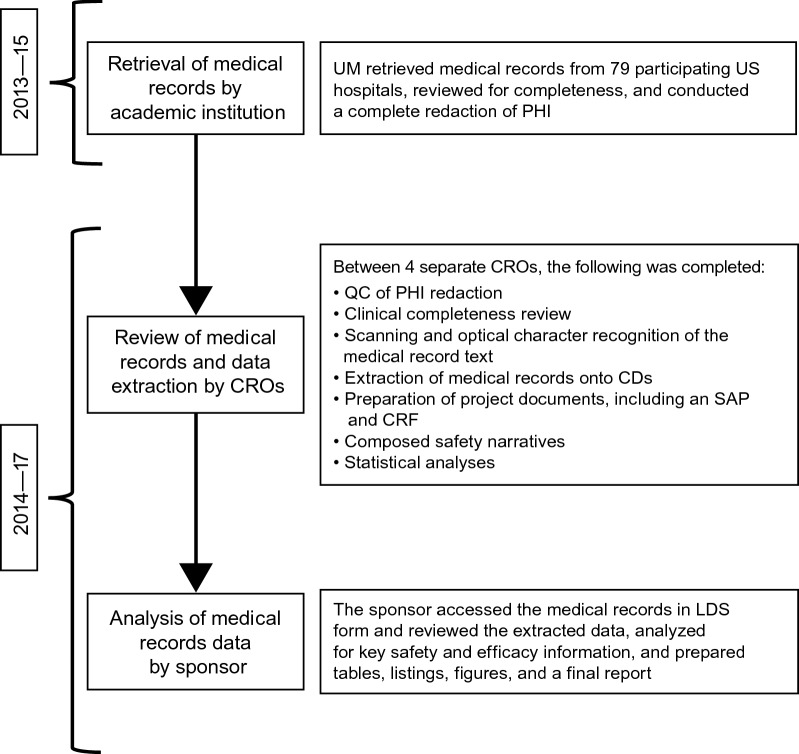
Medical records project (MRP) overview. *CD* compact disc, *CRF* case report form, *CRO* Contract Research Organization, *LDS* limited data set, *PHI* protected health information, *QC* quality control, *SAP* statistical analysis plan, *UM* University of Michigan

A collaborative partnership was established between the NDA sponsor (Pfizer, subsequently referred to as ‘the sponsor’), the academic institution that coordinated RAMPART (UM), US government agencies (NINDS at the NIH; Biomedical Advanced Research and Development Authority within the Department of Health and Human Services), and multiple contract research organizations (CROs; including Quintiles Inc, Medpace Inc, and MMS Holdings Inc). Each partner organization had a defined role under the program plan (Fig. 1).

This MRP was not classified as a clinical trial and had no testable hypothesis but was considered to more closely align with a non-interventional, retrospective chart review study. It was a unique program that was not aligned with any previously conducted internal studies or projects and therefore had had no specific internal processes and procedures to follow. As such, a dedicated program plan was developed, analogous to a clinical study protocol, which described the rationale, methodology, processes, procedures, and guidelines related to the implementation of the MRP. Where applicable, relevant aspects of the sponsor’s internal standard operating procedures for a non-interventional retrospective chart review study were leveraged, to ensure that the MRP was conducted in a robust manner, in order to support regulatory submission of the results. The program plan and subsequent amendments were reviewed and approved by an Institutional Review Board.

### Retrieval of Medical Records

UM retrieved the medical records of all RAMPART participants from the 79 participating US hospitals and assessed the completeness of each record. The initial review of each record (to ensure information required for patient enrollment was complete) was conducted by a clinical research professional supervised by a board-certified emergency physician. Any missing components were obtained by the respective hospital, if available.

UM also performed a complete redaction of personal information from each medical record prior to transferring the medical record to the sponsor. To ensure participant confidentiality, this redaction was performed by a dedicated team at UM in accordance with the Health Insurance Portability and Accountability Act requirements for a Limited Data Set. The redacted medical records, some of which exceeded 1000 pages, were transferred from UM to the NIH, and then to the sponsor and the CRO that conducted the data review, extraction, and databasing of the data, according to the project plan.

### Review of Medical Records and Data Extraction

All sections of each medical record were then fully reviewed by a CRO (conducted by licensed nurses and overseen by board-certified physicians) to identify the required data to be extracted, including all pertinent information recorded in the ambulance, in the ED, and during the entire in-patient hospitalization (if the participant was hospitalized), including the discharge summary. A case report form was created to collect the required medical record data to be extracted.

Data were extracted from the medical records according to the requirements pre-specified in the Program Plan (Table 1). Data were extracted from a medical record only reflecting the time period authorized by the participant’s signed informed consent document for the RAMPART study; if a patient withdrew consent or prematurely terminated participation in RAMPART, data were not extracted after consent withdrawal or premature termination of participation. However, for subjects who died, key data (e.g., cause of death, date of death) regarding the death that were present in public death records were included in the MRP database (but not the RAMPART analysis) even if it reflected the time period after decline of consent or study withdrawal.

**Table 1:** Summary of data extraction requirements

• Only verbatim information contained in RAMPART medical records that clearly met the data extraction definitions and requirements were extracted into the MRP database
• Assumptions or extrapolations of information from medical records were not permitted
• There was no attempt to compare or adjudicate any data discrepancies that may have existed between RAMPART data and the information extracted from medical records
• There was no contact with the hospital personnel who cared for participants, including the RAMPART investigator, to clarify data or request missing/incomplete data. UM reviewed each medical record to ensure that all requested components of the medical record were included prior to transmitting the medical record to the CRO. UM then followed up with the participating hospital to obtain any missing pages/data, and where possible, UM filled those gaps. After this process was completed, no further information was available
• During RAMPART, some participants were transferred directly from the ED of the participating hospital to a hospital that was not participating in RAMPART. Consequently, medical records from the non-participating hospital for those participants were not available for inclusion in the MRP database
• Day 0 was defined as the day of ED arrival
• While every attempt was made to read hand-written information in each medical record, only information that was legible could be reviewed and extracted. When necessary, multiple extractors would attempt to decipher information in a chart to gather information that was as complete as possible

*CRO* Contract research organization, *ED* emergency department, *MRP* medical records project, *RAMPART* Rapid anticonvulsant medication prior to ARrival trial, *SOP* standard operating procedure, *UM* University of Michigan

Four CROs were contracted to undertake different aspects of data extraction and the sponsor conducted extensive training with the personnel at each CRO on the required processes, procedures, and requirements of the MRP. The first CRO reviewed and extracted data from medical records into a secure electronic database, which was developed and validated via user acceptance testing procedures. If unredacted personal information was encountered in a medical record during review, the extraction was stopped immediately, the medical record was removed from the CRO’s internal medical record repository, and the sponsor was notified. The sponsor also removed the medical record in question from their internal medical record repository and immediately notified UM’s medical record redaction team. The medical record was then re-reviewed by UM’s medical record redaction team, as well as by a second CRO, to ensure that the medical record was fully redacted before data extraction was resumed.

The data extraction started with the date of the RAMPART participant’s enrollment and continued until the day the participant either withdrew consent, was discharged from the ED/hospital, or died. Electronic edit checks were created and defined in a data management plan, to ensure data quality. Data to be captured included safety-related events on Days 0, 1, and 2 of RAMPART, as that duration spans at least six half-lives for midazolam and lorazepam. Day 0 was defined as the day of the participant’s ED arrival. Events beginning after Day 2 through discharge (or death) were not considered temporally related to the administration of midazolam or lorazepam due to the short half-lives of these drugs. No clinical judgment, interpretation, or inference was made regarding the nature of the safety events captured from the medical records; only objective information documented in healthcare provider (HCP) notes was extracted.

Since the MRP was not a clinical trial, the safety events extracted from the medical records were not aligned with the standard definition of an AE, in contrast to the safety events that had been prospectively reported by the investigators during the RAMPART study. Safety data captured for the MRP were termed safety events (SEs) and serious safety events (SSEs) instead of AEs and SAEs. Despite this difference in terminology, and for consistency, the principles used to categorize safety data captured in the MRP database were based on the International Conference on Harmonisation (ICH) guideline definitions for AEs and SAEs (Table 2). In addition, safety events of interest (SEOI) were defined in the project plan and included renal, respiratory, and hepatic outcomes, as requested by the FDA.

**Table 2:** Definitions of adverse events

Safety events (SEs)	• Any untoward medical occurrence in the form of signs, symptoms, abnormal laboratory findings, or disease that started after the administration of RAMPART study treatments and/or worsened during the participant’s hospitalization • Abnormal laboratory findings were only considered an SE if the medical record included a clinical diagnosis or a statement regarding the abnormality. For example, a low hemoglobin value on a laboratory report alone was not entered as an SE, but if the medical record noted ‘anemia’ or ‘low hemoglobin,’ the verbatim term was entered as the SE
Serious safety events (SSEs)	• Any SE that: • Resulted in death. In case of a death, the cause of death was the SE, and the death was the outcome; • Was life-threatening. The term ‘life-threatening’ in this definition refers to an event in which the participant was at risk of death at the time of the event (including seizures as a medically important event); it does not refer to an event that hypothetically might have caused death if it were more severe; • Resulted in hospitalization or prolongation of existing hospitalization; • Resulted in persistent or significant disability/incapacity; or • Was a congenital anomaly/birth defect • Any adverse pregnancy outcome (e.g., spontaneous abortion, fetal death in utero, ectopic pregnancy, chronic fetal distress, stillbirth, neonatal death, or prematurity-related complication more than is typical for prematurity) was considered serious
Safety event of interest (SEOIs)	• Any event related to acute renal failure or acute central respiratory depression

The seriousness of an SE extracted from the medical records was determined based on the standard ICH guideline criteria for identifying SAEs. The severity of an SE (mild, moderate, or severe) was captured only if severity was explicitly stated in the medical record. For example, if the medical record stated ‘skin rash,’ with no documented indication of severity, then the SE would be captured as ‘skin rash’ and the severity would be recorded as ‘not available in the medical record.’ In general, the unavailability of data points was prospectively noted in the MRP database so that any subsequent requests for the data, from the FDA or other bodies, could be properly addressed.

The outcome of each SE was collected only if it was clearly documented in the medical record; otherwise, it was recorded as ‘not available in the medical record.’ No assessments of causal relationship to the study treatment were performed for individual SEs; if a relationship to drug was explicitly documented in the medical record, then that relationship was captured. If the SE was treated (pharmacologically or non-pharmacologically), then the details were recorded, when documented in the medical record. For each SE, the verbatim term was captured and then coded according to MedDRA version 18.1.

Preexisting medical conditions at the time of randomization were captured as medical history. However, if any preexisting condition was explicitly documented as having worsened in severity after randomization and study treatment administration, then that worsening was captured as an SE. Other pre-specified clinical information from each participant’s medical record was captured and stored in the database (Table 3).

**Table 3:** Pre-specified information collected from medical records

• Admitting diagnosis
• Intensive care unit admission and discharge date(s)
• Hospital admission history, including admission and discharge date(s)
• Discharge diagnosis/summary
• Deaths (if applicable)
• Autopsy report (if applicable and available)
• Physical examination results
• Vital signs (daily maximum and minimum):
• Blood pressure
• Heart rate
• Respirations
• Temperature
• Weight
Diagnostic test results
• Electrocardiogram reports and date(s) conducted
• Electroencephalogram reports and date(s) conducted
• Imaging (e.g., X-ray, computerized tomography, magnetic resonance imaging) reports and date(s) conducted
• Biopsy reports and date(s) conducted
• Laboratory test values (daily maximum and minimum, or single values as applicable; date(s) and times)
• Safety events, serious safety events, and safety events of interest (date(s), severity, seriousness, relationship to study treatment, and other information as available)
• Preexisting medical conditions
• Concomitant medications with date(s) of administration

The program plan specified the laboratory tests that were relevant to the pre-defined SEOIs previously described. These were collected in the MRP database, from Day 0 through ED/hospital discharge or withdrawal of consent, whenever the value had an identifiable unit documented in the medical records (Table 4). Local hospital reference ranges were used, whenever available, for designating a laboratory value as normal or abnormal. If no local reference range was available, then standard reference ranges were retrospectively applied, from either the FDA Investigations Operations Manual, or the National Library of Medicine laboratory test ranges. In addition to the targeted laboratory tests for which all values were captured, other abnormal laboratory test results were captured for Days 0, 1, and 2, but only the highest or lowest abnormal value for each day.

**Table 4:** Laboratory tests for SEs of interest collected from medical records

SE of interest	Laboratory test^a^	
Acute renal failure	Blood urea nitrogen^b^	
	Creatinine^b^	
Acute central respiratory depression	Blood carbon dioxide	Bicarbonate^b^ Carbon dioxide^c^
	Oxygen saturation^c^	
	Partial pressure of oxygen^c^	
	Arterial blood pH^c^	
Hepatic function	Alanine aminotransferase^b^	
	Alkaline phosphatase^b^	
	Aspartate aminotransferase^b^	
	Creatine phosphokinase^b^	
	Total bilirubin^b^	

*FDA* US Food and Drug Administration, *SE* safety event

^a^Daily maximum and minimum values, or single values as applicable, that were available in the medical records were captured and recorded in the MRP database

^b^Reference ranges were retrospectively applied from the FDA Investigations Operations Manual

^c^The National Library of Medicine laboratory test ranges were used as reference ranges

### Analysis

The database constructed for the MRP was used to summarize SEs and SSEs and, in conjunction with efficacy data from the RAMPART clinical study database, was used to perform additional analyses of pre-specified subpopulations.

The primary analysis population for summarizing the safety data collected from the RAMPART medical records included all participants randomized to midazolam or lorazepam (intent-to-treat population). In RAMPART and in the MRP, randomization was defined as occurring at the time the autoinjector was applied to the participant, prior to ED arrival. Participants were included in the primary analysis population even if study drug administration was not successful.

Due to the emergency setting in which RAMPART was conducted (pre-hospital treatment by emergency medical technicians; participants were always non-communicative), some participants were enrolled in the trial more than once (repeat enrollment). In these cases, data were extracted from the medical record for each separate enrollment.

The relationship between the primary efficacy outcome (odds of seizure termination before ED arrival without rescue medication) and study drug dose was determined by fitting a linear logistic regression model and modelling the log as a function of study drug dose (mg/kg). The relationships between the primary efficacy outcome and medical history and concomitant medications were qualitatively assessed by dividing participants into medical history and medication subgroups and comparing the percentage of participants achieving response between those who were and were not in each subgroup. For medical history, the subgroups were defined as patients with renal, respiratory, or hepatic impairment/disease history, or history of seizure. For concomitant medications, the subgroups were defined as patients who were taking opioids, benzodiazepines, enzyme inducers, enzyme inhibitors, enzyme inhibitors exclusive of inducers, and enzyme inducers exclusive of inhibitors.

For the primary analysis of SEs and SSEs, only the first enrollment was used for each participant. Additional analyses of SEs and SSEs were performed by (i) including only enrollments after the first, and (ii) by including all enrollments for each participant. SEs and SSEs were summarized according to treatment group with the denominator for each treatment group reflecting the number of participants who were randomized at least once to that treatment (i.e., some participants with more than one enrollment were randomized to both treatments).

## Results

**Fig. 2:**
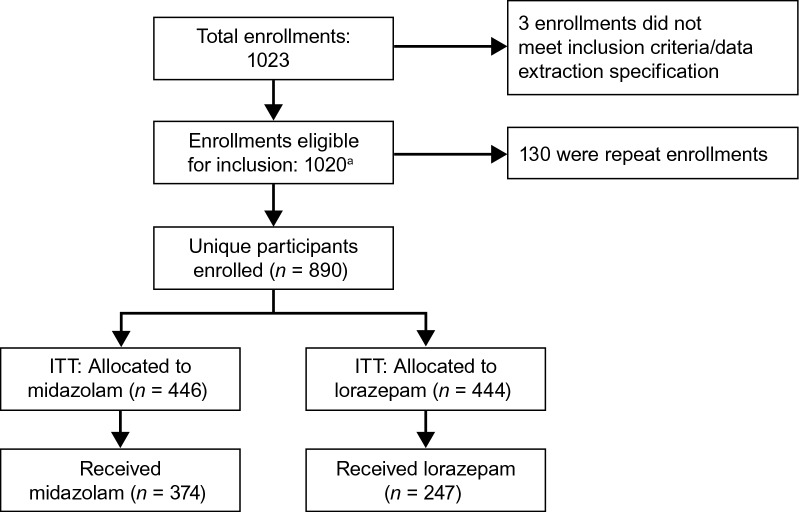
Patient disposition. This analysis of the ITT population utilized only the first enrollment for each participant. ^a^Includes *n* = 23 participants who withdrew consent after randomization and contributed partial data from the medical record. *ITT* intent-to-treat

Medical records abstracts were reviewed for 1020 hospitalizations of 890 individual RAMPART study participants (Fig. 2). Three additional medical records from RAMPART study participants were not included in this study as they (i) did not meet specifications for data extraction, or (ii) were not able to be obtained or reviewed.

The MRP database enabled a comprehensive assessment of SEs and SSEs that also included additional subgroup analyses based on comorbidities, including renal, respiratory, and hepatic impairment or disease, intracranial lesions, and seizure; and also analyses based on medication usage including enzyme inducers and inhibitors, opioids, benzodiazepines, respiratory depressants, and nephrotoxic agents.

The MRP database captured additional SEs and SSEs that were not within the AE reporting window, or were not interpreted as an SAE in the original RAMPART clinical study database. Based on MRP data, a total of 771 participants (86.6%) experienced an SE, and 495 (55.6%) had an SSE (Table 5), compared with 446 (49.9%) and 271 (30.3%) for AEs and SAEs, respectively, in the RAMPART clinical study database. The severity rating for most SEs and SSEs captured in the MRP database was missing (64.3%).

**Table 5:** Overview of SEs in the ITT population on Days 0, 1, and 2

*n* (%)	Midazolam (*n* = 446)	Lorazepam (*n* = 444)	Total (*N* = 890)
Any SE	379 (85.0)	392 (88.3)	771 (86.6)
SSEs^a^	231 (51.8)	264 (59.5)	495 (55.6)
Fatal events	9 (2.0)	4 (0.9)	13 (1.5)
Maximum severity			
Mild	53 (11.9)	68 (15.3)	121 (13.6)
Moderate	15 (3.4)	13 (2.9)	28 (3.1)
Severe	26 (5.8)	24 (5.4)	50 (5.6)
Missing	285 (63.9)	287 (64.6)	572 (64.3)

*ITT* intent-to-treat, *SE* safety event, *SSE* serious safety event

^a^SSEs that started on Days 0, 1, or 2

The original RAMPART database captured 20 deaths during the study: 11 in the midazolam arm and nine in the lorazepam arm. The MRP identified 22 deaths among RAMPART participants: 13 in the midazolam arm and nine in the lorazepam arm. The additional two deaths captured in the MRP database had not been included in the RAMPART study data because these events occurred after withdrawal of consent and discontinuation from the study. While assessment of the relationship of an SE or SSE to study drug was not performed in the MRP, the Sponsor did provide the FDA with a causality assessment for each death by considering the known adverse drug reactions for lorazepam and midazolam (e.g., acute respiratory failure, dyspnea, generalized tonic–clonic seizure, mental status changes, nausea, respiratory failure, and vomiting) and other factors including temporal relationship of the event to the drug. The MRP analysis concluded that one death was possibly related to study drug.

Based on analysis of the MRP safety database, no previously unrecognized adverse drug effects were identified for either midazolam or lorazepam. There was no significant relationship between the primary efficacy outcome (seizure termination before ED arrival without rescue medication) and study drug dose; and no association was observed between treatment success and any of the medical history or concomitant medication subgroups.

## Discussion

This MRP used unstructured, RWD from medical records to complement existing clinical trial data and facilitate FDA review and approval of an NDA, in September 2018, of intramuscular midazolam for the treatment of status epilepticus. The methods presented here may provide useful insights for others involved in drug development and attempting to meet similar regulatory submission requests.

This project involved a complex collaborative partnership between industry, academic, and government entities. The execution and success of the MRP was facilitated by the development and implementation of a comprehensive and robust strategy that clearly outlined the methodology, procedures, guidelines, communication, and mitigation strategies to be utilized.

In a typical non-interventional retrospective medical records review, the protocol targets a limited number of specific safety outcomes of interest. By contrast, this MRP project attempted to comprehensively capture, retrospectively, all relevant safety information from medical records in much the same way that a Phase 3 pivotal clinical trial aims to comprehensively capture all relevant AEs prospectively. This approach resulted in capturing a very large volume of safety and other data from the 1020 medical records reviewed from the RAMPART study, with the number of SEs and SSEs captured in the MRP project exceeding the number of AEs reported in the RAMPART trial, with the variance likely attributable to differing reporting windows and thresholds, and inability to clarify data with the investigators in the MRP as in the clinical trial database.

Moreover, an interventional clinical trial has an Investigator overseeing the care of the patient who will decide if a sign or symptom meets the definition of an AE and needs to be captured. The data reported to the clinical study database are organized and reviewed by the Principal Investigator prior to being submitted to a study sponsor, thus ensuring that the information is complete and internally consistent. A study sponsor can then query the investigator regarding potential data errors, missing data, or discrepancies identified during data review, and the investigator can resolve these data issues.

In the retrospective setting, that is not the case. Determining when an SE or SSE is, or is not, present in a medical record requires detailed rules to govern inference and instructions to aid the data extractors who will be reviewing and extracting the data from the medical records. In a retrospective review of medical records, there is no pathway to query an investigator or HCP involved in the patient’s care, in order to resolve data discrepancies or other issues, which can be limiting to the analysis. RWD does also have its limitations, which needs to be considered when designing a study. Per the FDA, to use RWD studies in support of regulatory decisions, these studies must still meet the legal requirements of an “adequate and well-controlled study” [12]. This includes a properly designed methodology and reliable, non-biased data sources. Due to the nature of retrospective data, it is possible the quality of data will not meet the FDA’s strict requirements. Additionally, it can be difficult to determine clinical interpretations of the data, leading to inclusion of non-relevant data or exclusion of relevant data.

With the recent increase in acceptance of RWD in support of regulatory decisions and the historic use of RWD for safety surveillance post-marketing, we believe that this MRP was an appropriate safety study to support the NDA and that other programs needing to supplement clinical trial data with additional safety data can utilize a similar process. However, the MRP differs from previously reported real-world studies. The hybrid model of data collection required for this project was highly complex, involving retrieval, review, and extraction of complete safety data from over 1000 medical records from 79 US hospitals. With no known precedent for this type of unstructured comprehensive data collection, this project required the development and implementation of a program plan to describe the detailed processes and procedures that would need to be followed to achieve the required deliverables.

## Conclusion

The MRP conducted for the RAMPART study represents a novel use of unstructured data collection from medical records to provide necessary supplemental safety information for FDA review of an NDA. During the initial stages of creating this study, FDA provided input in the data collection, verification, and analysis processes that was essential in designing this MRP study. The learnings from this project may be useful to others undertaking similar projects and/or studies that utilize RWD. Creative use of unstructured real-world retrospective medical record data can be considerably more efficient than conducting clinical trials, provided that the methodology includes well-designed processes, procedures, and guidelines for the comprehensive review, extraction, and analysis of the safety data in order to ensure acceptable quality, consistency, and robustness of the data and results. Finally, this MRP demonstrated that a collaborative partnership between pharmaceutical industry sponsors, academic institutions, US government agencies, and CROs can be effective and successful.
